# Supplementary material for: WRKY Gene Family Drives Dormancy Release in Onion Bulbs
Source: Cells. 2022 Mar 24;11(7):1100. doi: 10.3390/cells11071100 (PMC8997782; doi:10.3390/cells11071100)

Supplementary material

Tables

Table S1 Primer list used in qPCR for 14 *AcWRKYs* validation. ID, gene's name, primer sequences and expected size for each gene are reported in the table.

Gene ID/reference	Name	Primer	Sequences (5' – 3')
TRINITY_DN15477_c0_g1	*AcWRKY1*	Forward	GGGTACGGGTTCCAGATCTG
		Reverse	ACCCCACTATGCTGGTTGTG
TRINITY_DN24552_c0_g2	*AcWRKY2*	Forward	CACTTCTCCGTCACCCATGT
		Reverse	ACGAAGCAAGAATCATCAAAGCA
TRINITY_DN22804_c0_g1	*AcWRKY3*	Forward	TGAACTCTTACCTGCCGCAG
		Reverse	GCAACCCATGATCCCTGACA
TRINITY_DN33211_c0_g1	*AcWRKY9*	Forward	AACTCGACCTCAGCGTTGTT
		Reverse	GTCGCCCAAATTCACCATTGA
TRINITY_DN1383_c0_g1	*AcWRKY12*	Forward	AGCTACTACAGATGCACGCA
		Reverse	TGGACTTCTGAATAGGATGAGTGT
TRINITY_DN6283_c1_g1	*AcWRKY17*	Forward	ACCTCCTTTTACATTGGAAGTGC
		Reverse	ACGAATTCACAAAAGAGTCCCCT
TRINITY_DN9124_c1_g1	*AcWRKY18*	Forward	TCCCTTTCAACTGCTCGTCG
		Reverse	TCTCTTCAAACTCGAACTGCCA
TRINITY_DN10137_c1_g1	*AcWRKY21*	Forward	ATGCTCTTTTGCCCCACTGT
		Reverse	CGTGTATGTGCTCACCTTCG
TRINITY_DN8131_c0_g1	*AcWRKY26*	Forward	TCCTGAACGGTTGTCATGCA
		Reverse	CCGGTTTTATTCAAGTCCACTCC
TRINITY_DN32880_c0_g1	*AcWRKY30*	Forward	AGCCTGCACATCGAAACTCT
		Reverse	GGAACTCCGATCAAGGTTGGT
TRINITY_DN15141_c0_g1	*AcWRKY32*	Forward	CGCTGATCCTAATTTCACGGC
		Reverse	ATTGTTGTTGGCACCATTACCA
TRINITY_DN24099_c0_g1	*AcWRKY37*	Forward	TGCCGAACTTGTATCTTCCCA
		Reverse	CGGTGGAGGAAATATGGGCA
TRINITY_DN13617_c0_g1	*AcWRKY46*	Forward	CAAACGCTTCCACCTACAAACA
		Reverse	TCTTGATCGTTGCATGAGAAGTC
TRINITY_DN6283_c0_g1	*AcWRKY52*	Forward	AGAAATGTTCCAAGGCTCGGA
		Reverse	TCCGTGTTCTAGTTCTATGGTACAAA
ADC83988.1 (Khosa et al., 2016)	*Actin*	Forward	CTGGGATGACATGGAGAAGATT
		Reverse	GTTAAGTGGAGCCTCCGT

Table S2 ABA concentration (ug/g) in uninfected and OYDV-infected samples at each sampling time (T_1, T_2, and T_3) evaluated by HPLC/MS.

Time	Sample	ABA (ug/g)	St. dev.
T1	Uninfected	0.995	0.147
	OYDV-infected	0.376	0.097
T2	Uninfected	0.269	0.001
	OYDV-infected	0.579	0.004
T3	Uninfected	0.197	0.096
	OYDV-infected	1.311	0.179

Table S3 Overview of sequencing and assembly output of *A. cepa* bulb transcriptome.

**Items**	**Statistics**
Number of contigs	581,029
Number of sequences after cd-hit tool	479,145
Total length (nt) of transcripts	440,954,936
Total number of genes	316,298
Longest sequence (nt)	15,759
Mean sequence length (nt)	920
Median sequence length (nt)	494
N50 sequence length (nt)	1,615
GC-content (%)	36.72
BUSCO Complete	87.71%
BUSCO Complete + Partial	91.88%
BUSCO Missing	8.12%

Table S4 Summary of DEGs (Padj < 0.05) extracted between uninfected and OYDV infected samples at T_2/T_1, T_3/T_1, and T_3/T_2 comparisons. The total number of DEGs, up (Log2 fold change > 1) and down (Log2 fold change < -1) regulated genes were indicated

**Sample**	**Comparison**	**Number of DEGs**	**Log2 fold change > 1**	**Log2 fold change < -1**
Uninfected	T_2/T_1	1,253	325	796
	T_3/T_1	2,278	1,129	861
	T_3/T_2	1,859	1,326	362
	Total	*5,390*	*2,780*	*2,019*
OYDV-infected	T_2/T_1	304	100	189
	T_3/T_1	401	191	184
	T_3/T_2	617	319	248
	Total	*1,322*	*610*	*621*

Table S5 List of annotated DEGs, and their fold change (log2 Fold-Change), isolated at T_2/T_1, T_3/T_1, and T_3/T_2, comparisons, in uninfected and OYDV-infected bulbs using MapMan software. Fold change and annotation is reported for each gene.

Table S6 List of annotated DEGs, and their fold change (log2 Fold-Change), isolated at T_2/T_1, T_3/T_1, and T_3/T_1, comparisons, in uninfected and OYDV-infected bulbs using MapMan software. Fold change and annotation is reported for each gene. The present list has been used to develop Figure 2.

Table S7 List of onion *AcWRKYs* isolated.

Table S8 Summary of *AcWRKY* DEGs detected in both uninfected and OYDV-infected bulbs in each comparison

**Condition**	**T_2/T_1**	**T_3/T_1**	**T_3/T_2**
Uninfected	*WRKY18, 21 37*	*WRKY2, 9, 12, 17, 30, 32, 46, 52*	*WRKY1, 2, 3, 9, 12, 17, 18, 21, 26, 37, 46, 52*
OYDV-infected	-	-	-

Table S9 RNASeq (log2 FC) and qPCR ($2^{-\Delta\Delta Ct}$) values for *AcWRKYs* differentially expressed in each comparison, isolated in uninfected samples, were indicated. Expression pattern of *AcWRKYs* extracted were also evaluated for uninfected (U)/OYDV-infected (I) bulbs comparison at T_3. Genes showing significant differences ($p<0.05$) between conditions (U and I) by RNASeq and qPCR were underlined. For each *AcWRKY* is reported the transcript ID and its orthologous in *A. thaliana* (*AtWRKY*).

ID	*AcWRKY*	*AtWRKY*	T_2/T_1		T_3/T_1		T_3/T_2		T_3 U/I	
			RNASeq	qPCR	RNASeq	qPCR	RNASeq	qPCR	RNASeq	qPCR
TRINITY_DN15477_c0_g1	*AcWRKY1*	*AtWRKY69*	-	-	-	-	1.026	2.126	-	-
TRINITY_DN24552_c0_g2	*AcWRKY2*	*AtWRKY41*	-	-	1.988	2.744	3.131	4.321	1.101	3.745
TRINITY_DN22804_c0_g1	*AcWRKY3*	*AtWRKY71*	-	-	-	-	1.163	2.662	0.845	1.921
TRINITY_DN33211_c0_g1	*AcWRKY9*	*AtWRKY46*	-	-	1.410	1.314	2.133	3.988	-	-
TRINITY_DN1383_c0_g1	*AcWRKY12*	*AtWRKY75*	-	-	1.522	2.056	1.408	2.901	-	-
TRINITY_DN6283_c1_g1	*AcWRKY17*	*AtWRKY26*	-	-	1.008	1.638	1.974	3.208	-	-
TRINITY_DN9124_c1_g1	*AcWRKY18*	*AtWRKY67*	-2.155	-2.976	-	-	3.604	5.150	-	-
TRINITY_DN10137_c1_g1	*AcWRKY21*	*AtWRKY40*	-2.288	-2.325	-	-	3.047	3.354	-	-
TRINITY_DN8131_c0_g1	*AcWRKY26*	*AtWRKY51*	-	-	-	-	2.685	4.836	-	-
TRINITY_DN32880_c0_g1	*AcWRKY30*	*AtWRKY22*	-	-	3.773	9.114	-	-	4.831	5.001
TRINITY_DN15141_c0_g1	*AcWRKY32*	*AtWRKY6*	-	-	2.491	7.681	-	-	1.937	1.535
TRINITY_DN24099_c0_g1	*AcWRKY37*	*AtWRKY70*	-1.297	-1.186	-	-	1.634	3.335	-	-
TRINITY_DN13617_c0_g1	*AcWRKY46*	*AtWRKY55*	-	-	3.477	8.017	4.425	8.202	-	-
TRINITY_DN6283_c0_g1	*AcWRKY52*	*AtWRKY33*	-	-	2.441	2.397	3.315	4.454	-	4.856

Table S10 List of genes extracted in Co-expression network analyses by using as baits 3 *AcWRKYs* up-regulated at T_3 in the uninfected/OYDV infected bulbs comparison (*AcWRKY2*, *30*, *32*).

Figures

Figure S1 Rooting evaluation between **A)** uninfected and **B)** OYDV-infected bulbs across sampling time. Percentage is referred to the number of onion which show rooting at the three selected time points.

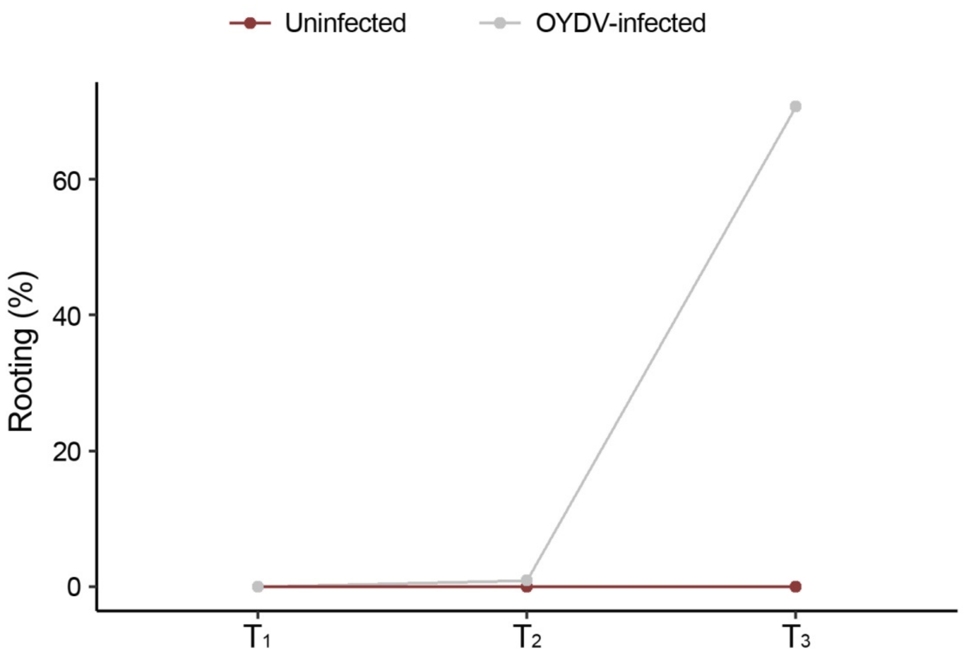

Figure S2 GO terms enrichment analysis carried out at each sampling times investigated in uninfected and OYVD-infected bulbs. A) Biological Process (BP); B) Molecular Function (MF); C) Cellular Component (CC).

A)

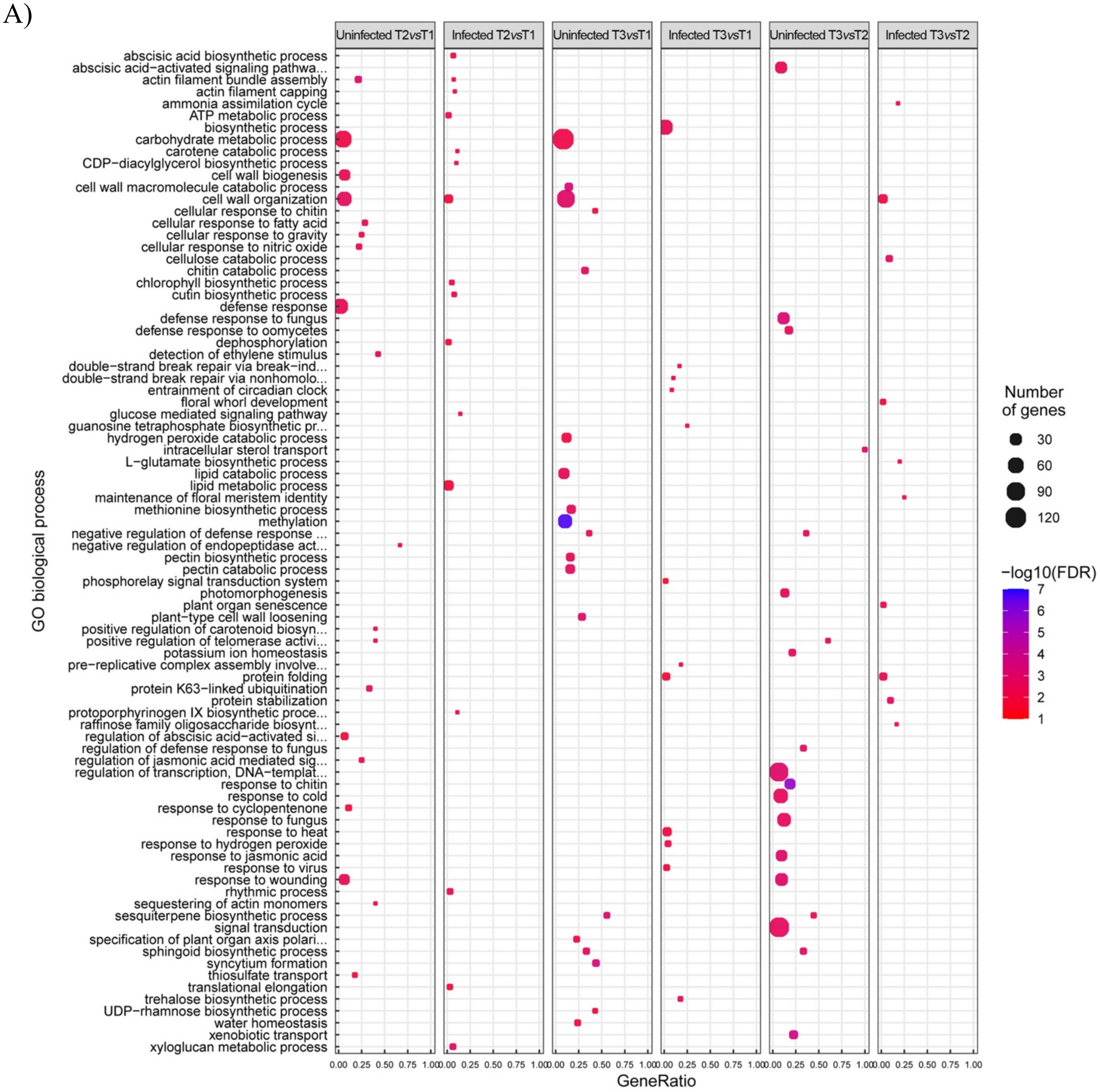

B)

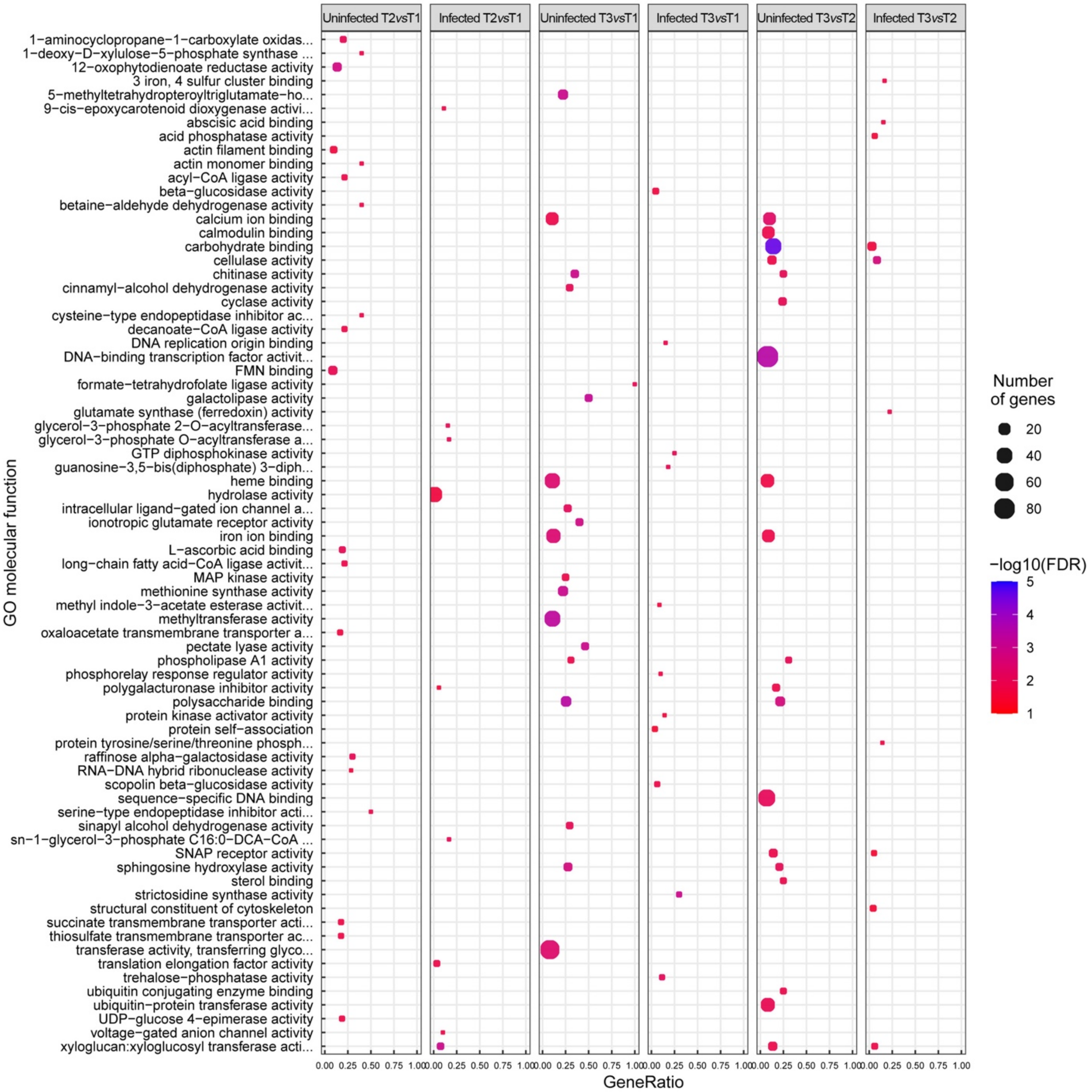

Uninfected T2vsT1
Infected T2vsT1
Uninfected T3vsT1
Infected T3vsT1
Uninfected T3vsT2
Infected T3vsT2
GO molecular function
GeneRatio
Number of genes
20
40
60
80
−log10(FDR)
5
4
3
2
1

C)

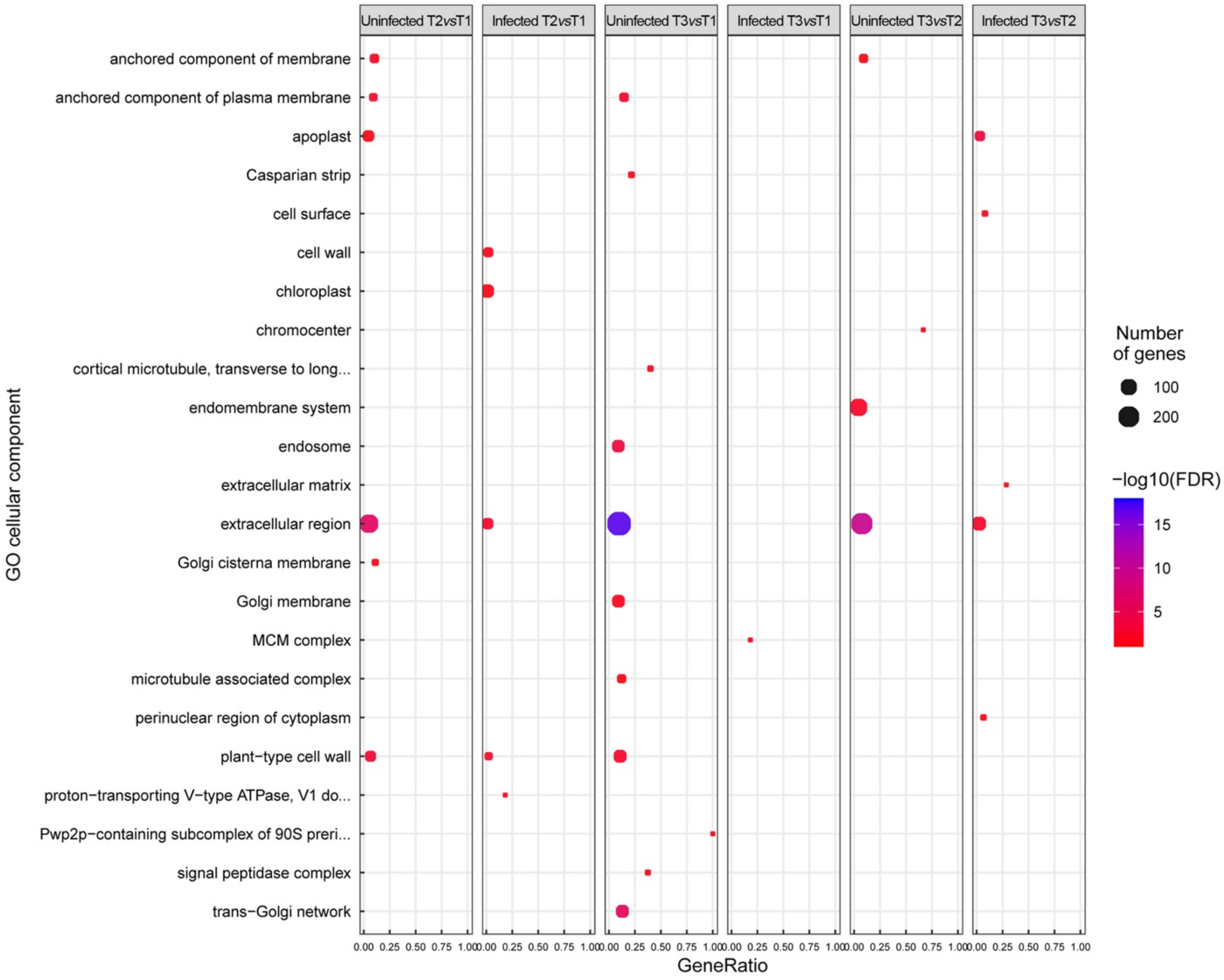

Uninfected T2vsT1
Infected T2vsT1
Uninfected T3vsT1
Infected T3vsT1
Uninfected T3vsT2
Infected T3vsT2
GO cellular component
anchored component of membrane
anchored component of plasma membrane
apoplast
Casparian strip
cell surface
cell wall
chloroplast
chromocenter
cortical microtubule, transverse to long...
endomembrane system
endosome
extracellular matrix
extracellular region
Golgi cisterna membrane
Golgi membrane
MCM complex
microtubule associated complex
perinuclear region of cytoplasm
plant−type cell wall
proton−transporting V−type ATPase, V1 do...
Pwp2p−containing subcomplex of 90S preri...
signal peptidase complex
trans−Golgi network
0.00 0.25 0.50 0.75 1.00
GeneRatio
Number of genes
100
200
−log10(FDR)
15
10
5

Figure S3 Transcriptome relationships among the three time points during onion bulb dormancy of uninfected and OYDV-infected plants. VENN diagrams of transcriptome profiles at T_2/T_1 (**A**), T_3/T_1 (**B**), and T_3/T_2 (**C**) comparisons. PCA (**D**) developed using five hundred most variable genes.

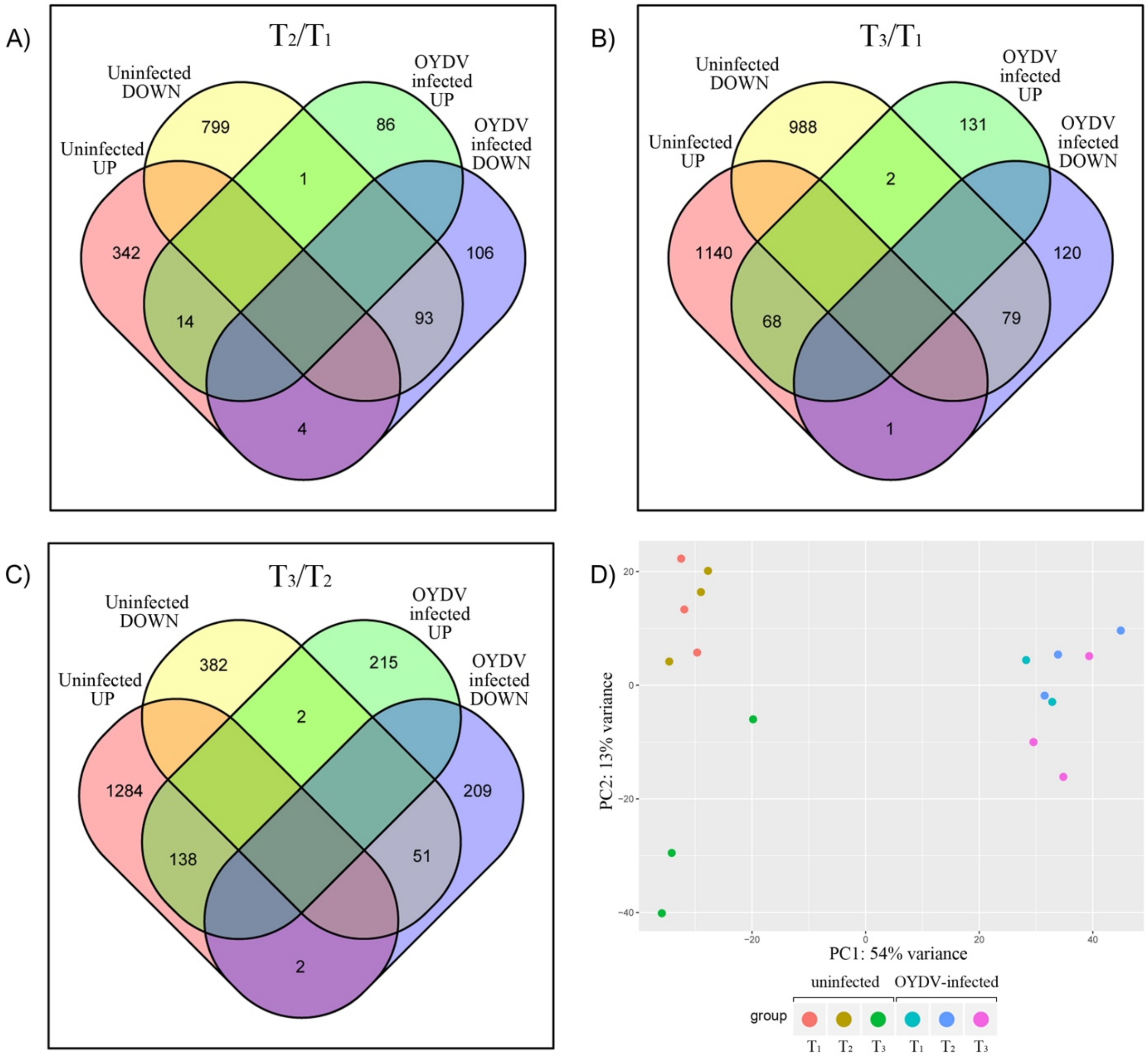

Figure S4 Overview expression level changes of genes associated to hormone signaling, and some key metabolism pathways involved in dormancy process between uninfected and OYDV infected, following the Mercator annotation. **A)** T_2/T_1, **B)** T_3/T_1, and **C)** T_3/T_2 comparisons. Transcription levels with a value log2 FC ≥ 1 for pairwise comparisons are displayed in colored squares. The blue and red indicate up- and down-regulated genes, respectively. The scale showing the expression level is displayed he scale showing the expression level is displayed in the pictures that have been exported from MapMan and their quality was further improved accordingly.

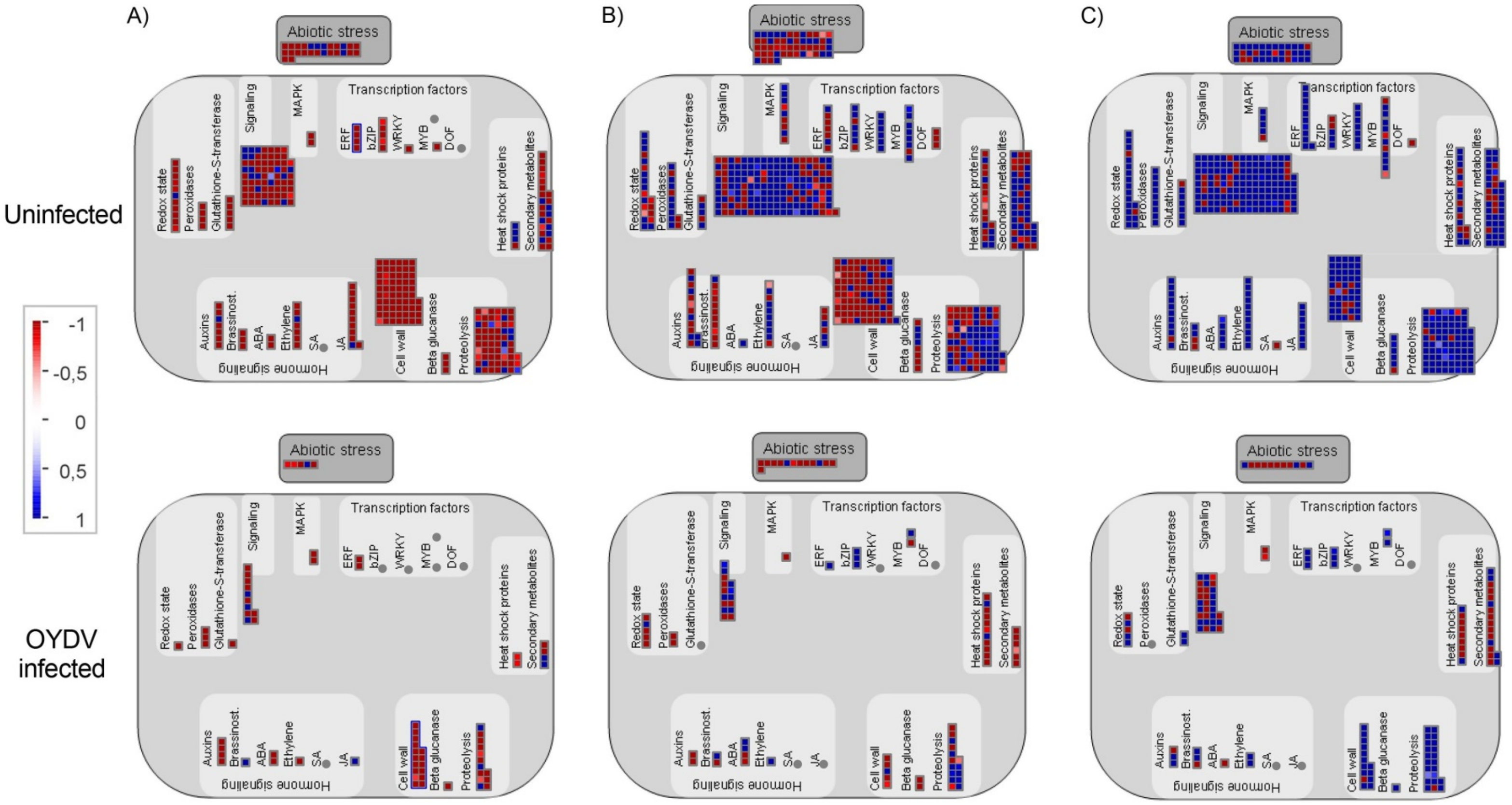

Figure S5 Phylogenetic tree of deduced *A. cepa* WRKY genes and their structural organization. The groups were highlighted through different colors. The sequence of each conserved motif is displayed on the bottom. Each motif was drawn to scale.

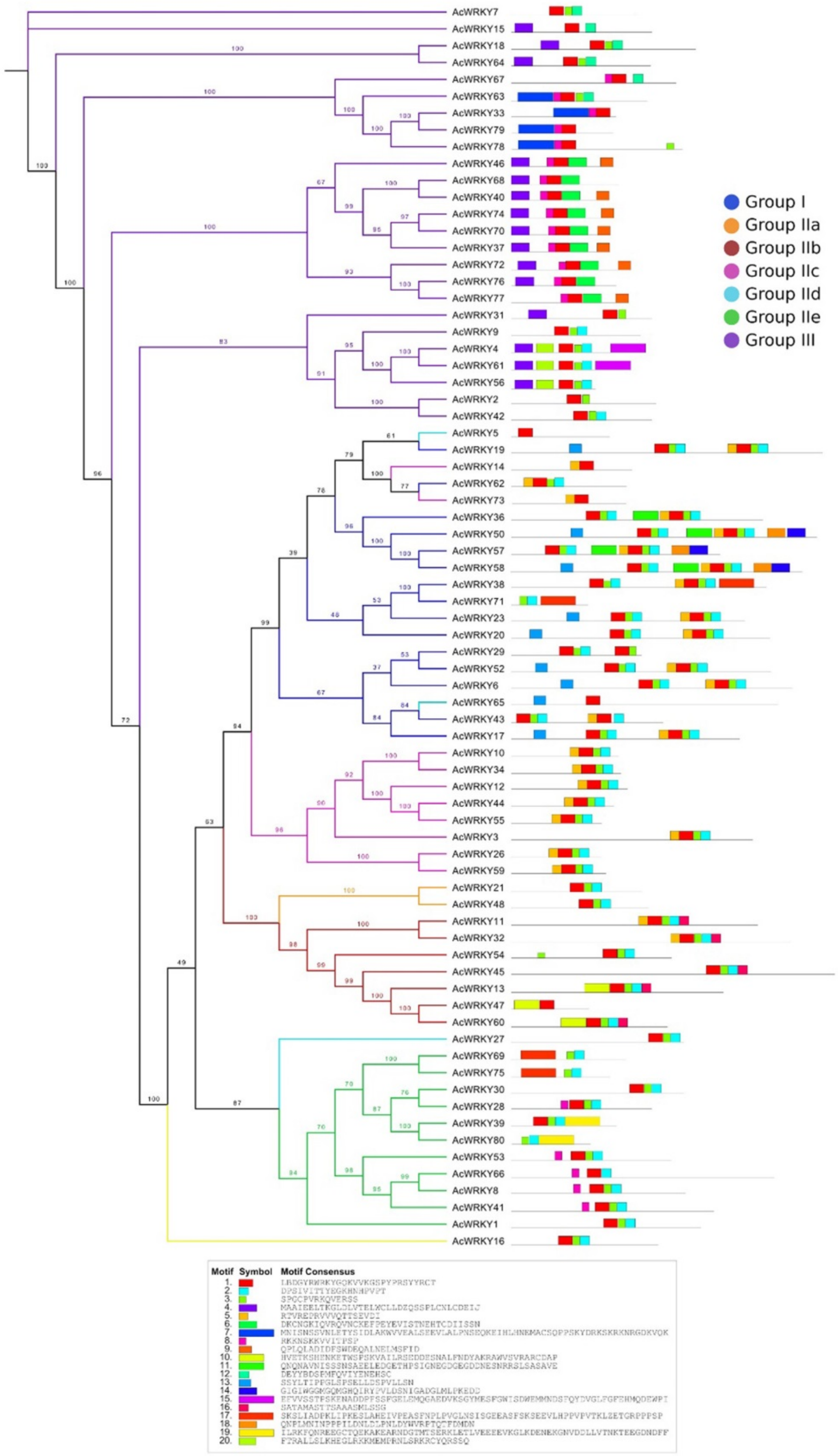

Figure S6 Correlation analysis between RNA-Seq and qPCR data for *AcWRKYs* differentially expressed in each comparison (Table S9). The same RNAs from NGS experiment were utilized for validation. The Pearson Correlation Coefficient (R = 0.88) and the p-value (p = 1e-09) were reported.

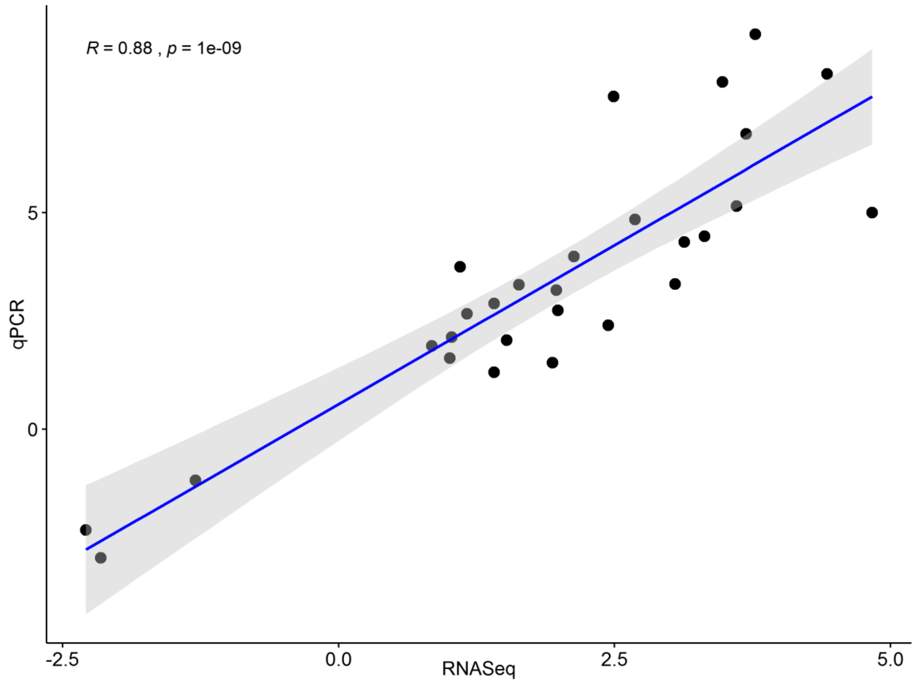

Figure S7 Flow chart of the experimental procedure carried out in the present study.

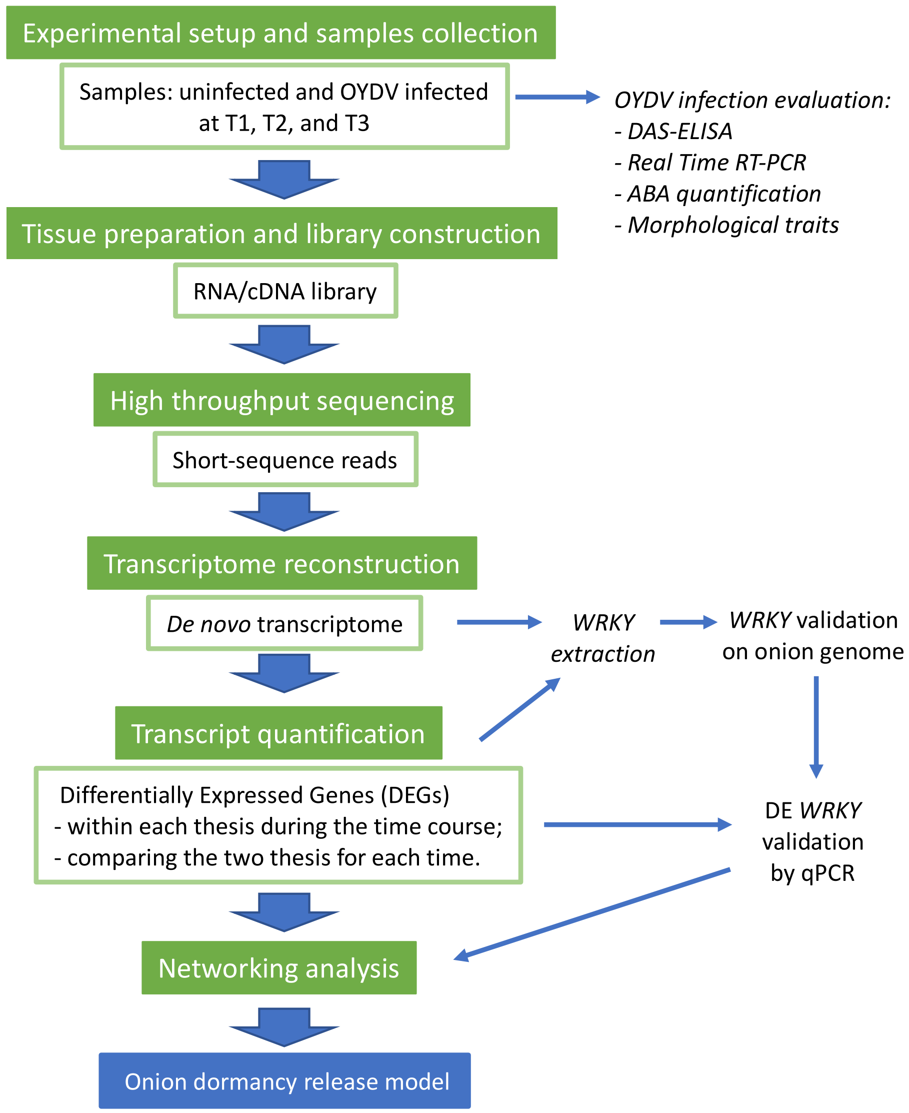

Supplement: Supplementary file 1 [file cells-11-01100-s001.zip › Puccio_et_al_CELLS_SUPP_MAT_R1.pdf]
